# A Systematic Review and Meta-analysis of Convolutional Neural Network in the Diagnosis of Colorectal Polyps and Cancer

**DOI:** 10.5152/tjg.2023.22491

**Published:** 2023-10-01

**Authors:** Kamyab Keshtkar, Ali Reza Safarpour, Ramin Heshmat, Rasoul Sotoudehmanesh, Abbas Keshtkar

**Affiliations:** 1 University of Tehran School of Electrical and Computer Engineering, Tehran, Iran; 2 Gastroenterohepatology Research Center, Shiraz University of Medical Sciences, Shiraz, Iran; 3 Chronic Diseases Research Center, Endocrinology and Metabolism Population Sciences Institute, Tehran University of Medical Sciences, Tehran, Iran; 4 Department of Gastroenterology, Digestive Disease Research Center, Digestive Disease Research Institute, Tehran University of Medical Sciences, Tehran, Iran; 5 Department of Health Sciences Education Development, Tehran University of Medical Sciences School of Public Health, Tehran, Iran

**Keywords:** Convolutional neural networks, colorectal polyps, colorectal cancer, computer-aided diagnosis

## Abstract

**Background/Aims:**

Convolutional neural networks are a class of deep neural networks used for different clinical purposes, including improving the detection rate of colorectal lesions. This systematic review and meta-analysis aimed to assess the performance of convolutional neural network–based models in the detection or classification of colorectal polyps and colorectal cancer.

**Materials and Methods:**

A systematic search was performed in MEDLINE, SCOPUS, Web of Science, and other related databases. The performance measures of the convolutional neural network models in the detection of colorectal polyps and colorectal cancer were calculated in the 2 scenarios of the best and worst accuracy. Stata and R software were used for conducting the meta-analysis.

**Results:**

From 3368 searched records, 24 primary studies were included. The sensitivity and specificity of convolutional neural network models in predicting colorectal polyps in worst and best scenarios ranged from 84.7% to 91.6% and from 86.0% to 93.8%, respectively. These values in predicting colorectal cancer varied between 93.2% and 94.1% and between 94.6% and 97.7%. The positive and negative likelihood ratios varied between 6.2 and 14.5 and 0.09 and 0.17 in these scenarios, respectively, in predicting colorectal polyps, and 17.1-41.2 and 0.07-0.06 in predicting colorectal polyps. The diagnostic odds ratio and accuracy measures of convolutional neural network models in predicting colorectal polyps in worst and best scenarios ranged between 36% and 162% and between 80.5% and 88.6%, respectively. These values in predicting colorectal cancer in the worst and the best scenarios varied between 239.63% and 677.47% and between 88.2% and 96.4%. The area under the receiver operating characteristic varied between 0.92 and 0.97 in the worst and the best scenarios in colorectal polyps, respectively, and between 0.98 and 0.99 in colorectal polyps prediction.

**Conclusion:**

Convolutional neural network–based models showed an acceptable accuracy in detecting colorectal polyps and colorectal cancer.

**Main Points**

- Convolutional neural network–based models could have an acceptable accuracy in detecting polypoid lesions and colorectal malignancies.
- The performance of these models would require an adequate training dataset.
- Unfortunately, available studies on the diagnostic performance of convolutional neural network—based models in colon polyps and cancer have low methodologic quality.

## Introduction

Colorectal cancer (CRC) is the third most common cancer and the second cause of cancer-related deaths worldwide.^1^ Although the biological pathways that transform the normal colon tissue into malignant tissue are different, polyps are presumed to be the precursor lesions for malignant tumors in all cases.^2^ Colorectal polyps (CRP) can be seen in various forms or shapes on colonoscopy. Based on their growth pattern, CRP are histologically classified as hyperplastic polyps or adenomatous (adenomas) polyps.^2^

A new systematic review and meta-analysis on 70 primary population-based cross-sectional studies that used colonoscopy for assessing different colorectal neoplastic lesions reported the worldwide prevalence of adenoma, advanced adenoma, and CRC in patients older than 50 years as 25.9%, 5.2%, and 0.6%, respectively.^3^ Brenner et al^4^ investigated almost 850 000 colonoscopies from the national CRC screening program in Germany and concluded that the 10-year risk of progression of advanced adenomas to CRC varied between 25% and 40% in men and women older than 50 years of age.

Screening colonoscopy, as an effective strategy for the prevention of CRC development, can decrease CRC mortality by both polypectomy/adenoma removal and detecting CRC at its early stages.^5^ Since CRP removal does not necessarily eliminate the risk of CRC, current guidelines recommend a 5-10-year rescreening interval after a negative colonoscopy result.^6,7^ Recently, a large-scale multi-center cohort study followed 200 000 persons who underwent baseline colonoscopy for over 8 years and showed that high- and low-risk adenomas increased the risk of CRC development by respectively 2.6 and 1.3 folds when compared to persons without adenoma.^8^

Despite the efforts in the screening CRC/CRP in target groups (e.g., individuals over 50 years of age) during the past 2 decades, 25% of adenomas or pre-cancerous lesions may be missed in conventional colonoscopies even if performed by expert gastroenterologists.^9,10^ Over the past few decades, several measures have been developed and recommended to evaluate the quality of colonoscopy for the diagnosis of polyps and colon cancers.^11^ An important quality measure is the adenoma detection rate (ADR). A recent large-scale investigation on more than 300 000 colonoscopies performed by 136 colonoscopists during 13 years showed that a 1.0% increase in the ADR was associated with a 3.0% decrease in the risk of CRC.^12^

In recent years, the combination of various technologies with colonoscopy has led to new endoscopic methods and improved detection rate of colorectal lesions. Image-enhanced endoscopy, cap-assisted colonoscopy, the Third Eye® Retroscope®, wide-angle colonoscope, Endocuff® device, and water-assisted colonoscopy are just a few of these new methods of colonoscopy that are available to augment the detection, diagnosis, and treatment rates of these subtle lesions.^13,14^

In the past few years, several adjunct techniques or devices, called “computer-aided diagnosis” (CAD), have been under investigation for improving ADR in colonoscopy settings.^15^ Various models such as “artificial intelligence” (AI), “deep neural networks,” and “machine learning” are categorized under CAD. Recent investigations have shown that CAD methods, along with colonoscopy data, have advantages including higher CRC/CRP detection, better histopathologic differentiation, decreased overall healthcare costs, and decreased operator dependency.^15^

Convolutional neural networks (CNNs) are a class of deep neural networks highly effective at performing image and video analysis. Computer-aided diagnosis—convolutional neural network models for colonoscopy could assist endoscopists in detecting polyps and performing optical diagnosis. Convolutional neural networks are trained by using thousands of colonoscopy images to detect hyperplastic and adenomatous polyps and differentiate them.^16,17^

Since 2018, several systematic reviews have focused on the diagnostic value of CNN methods in different clinical conditions including skin cancer,^18^ breast cancer,^19^ hepatocellular carcinoma or hepatic mass,^20^ and ischemic brain strokes.^21^

As primary studies assessing the diagnostic value or predictive performance of AI models compared to conventional/standard colonoscopy techniques were mainly conducted and published after 2016, secondary studies or systematic reviews in this field are quite recent. In fact, systematic reviews and meta-analyses comparing conventional or standard colonoscopy techniques with techniques based on AI algorithms applied on colonoscopy images or videos were only published in 2020 and 2021.^22–35^

The majority of these systematic reviews did not use diagnostic indicators [sensitivity, specificity, positive predictive value (PPV), and negative predictive value (NPV), etc.] to combine primary studies and instead included odds ratio (OR) or relative risk (RR) indices to combine studies. Only 3 systematic reviews^26,27,33^ used the appropriate approach of combining these primary studies and reported diagnostic indicators.

The main challenges of the previous systematic reviews were a lack of comprehensive search (including search in conference papers or proceedings) and using inappropriate risk of bias assessment checklist [the appropriate RoB tool for prediction model studies is the Prediction model Risk of Bias Assessment Tool (PROBAST) checklist^36^].

Therefore, in the present systematic review, a comprehensive search was performed in various scientific sources (even papers presented at conferences) in different fields including medicine, engineering, and computer science, and the proper approach to systematic reviews of predictive models was adopted to assess the performance of CNN-based models in detection or classification of polyps or malignant colorectal lesions.

## Materials and Methods

The protocol of this study was first designed based on the “priori” (or pre-defined status) approach^37^ and then registered in the “Open Science Framework” (OSF) using open-ended format and CC0 license. The protocol has a DOI (10.17605/OSF.IO/QJ7EU) and is publicly accessible at https://osf.io/qj7eu. Furthermore, the study protocol has been published.^38^

## Eligibility Criteria of Primary Studies

### Types of Primary Studies

Primary studies were included if they adopted a cross-sectional, prospective, or retrospective design and recruited subjects who had colonoscopy videos or images which showed pathological or cytological diagnoses (as the gold standard or reference standard). Therefore, all observational studies with case-control, cohort, or cross-sectional designs were included. Interventional studies (trials, experimental, or quasi-experimental), reviews (secondary research), editorials, letters, and similar articles were excluded.

### Types of Participants

Since CRPs are usually diagnosed in adults older than 50 years of age, all studies conducted on adult populations of either gender (age >18 years) were eligible to be included.

### Reference Standard

The histopathological examination results of the colorectal lesions (CRC or CRP) were used as the reference standard. The histological data were confirmed by expert histo/pathologists.

### Index Test (the Output of the Prediction Model)

Convolutional neural networks are a supervised learning method. They can learn and find the relationship between the input (images or videos) and the class labels. Convolutional neural network layers are generally divided into 2 categories: convolutional and pooling layers (hidden layers) and fully connected layers. The task of the hidden layers is to extract the features. The fully connected layers are used for the classification and detection of objects in the input images at the end of the CNN. The different class labels of all the assessed images or videos should be reported.

### Search Strategy

We searched the following bibliographic databases: PubMed/MEDLINE, SCOPUS, Web of Science, IEEE (Institute of Electrical and Electronics Engineers), Inspec, ProQuest, Google Scholar, Microsoft Academic Search, ScienceOpen, arXiv, and bioRxiv. Moreover, we assessed relevant conferences for content (Conference on Computer Vision and Pattern Recognition, International Conference of Computer Vision, European Conference on Computer Vision). We hand-searched *Gastroenterology*, *Pattern Recognition*, *Scientific Reports* as key journals. The publication time was limited from January 1, 2010, to July 31, 2020. The search was not restricted based on language or geographical area. The PubMed search syntax is shown in Supplementary Box 1.

### Screening and Selection Processes

After searching the mentioned sources, AK and ARS screened all the primary studies based on titles or abstracts. A screening checklist was developed using 46 criteria. The criteria were selected based on the most common components reported in the abstracts of primary research. We selected the eligible or potentially eligible studies for further assessment. AK and ARS independently evaluated the considered studies based on full-text papers or documents and resolved any disagreement by consensus or a third reviewer (KK).

### Quality Assessment (Risk of Bias Assessment)

ARS and RH independently assessed the ROB of the included studies. The ROB checklist was developed based on PROBAST.^36^ The checklist had 2 main domains including ROB and applicability domains. The ROB domain contained 4 items: participants, predictors, outcome, and analysis. The applicability domain had 3 items: participants, predictors, and outcomes. All 7 items were rated as low ROB, high ROB, and unclear ROB. The overall quality status (overall ROB status) was determined based on the defined guidelines (PROBAST guideline). Any disagreements were resolved by consensus or a third reviewer (KK).

### Data Extraction and Data Synthesis

We designed an extraction form based on the study objectives and finalized it after its testing on at least one study. AK and KK independently extracted the required data from all the primary studies (papers or documents) and resolved any disagreements in the extracted data through consensus.

There were 2 primary measures of predictive performance including the area under curve–receiver operating characteristic curve (AUC–ROC; C or concordance statistics) and the other performance measures [sensitivity, specificity, PPV, NPV, positive likelihood ratio (PLR), negative likelihood ratio (NLR), accuracy and diagnostic OR (DOR)]. As primary data synthesis (meta-analysis), we combined the AUC and/or the other performance measures.

Data of the secondary objectives were the CNN architecture model, e.g., VGG,^39^ AlexNet,^40^ and GoogleNet,^41^ or the features of the CNN architecture, such as the number of layers, the size of layers [kernel (filter) and stride size], and the kind of pooling layers (max or average), the CNN model sensitivity, specificity, and DOR. The subgroup variable data would be transfer learning (existing or absent), learning rate, and the features of CNN architecture (e.g., number of layers and size of the kernel and stride).

### Statistical Analysis

Combining data of the primary and secondary objectives was performed based on the guidelines provided by Debray et al.^42^ Stata 14.2 (StataCorp. College Station, TX, USA) and R 4.0.0 were used for conducting the meta-analysis.

A forest plot was used for presenting the performance measure pooling and inconsistency (I^2^) measure, and Cochran’s Q test was applied for heterogeneity assessment.^43^ Subgroup analysis or meta-regression was also conducted based on the abovementioned variables to determine the potential sources of heterogeneity. The funnel plot, Begg’s or Egger’s tests,^44^ and fill and trim methods^45^ were used to evaluate publication or reporting bias. As a sensitivity analysis, the leave-one-out method was also applied to assess the relationship between the primary research quality and the overall accuracy.^46^

Since primary studies developed different CNN models with different performance measures for each of the CRP and CRC outcomes, not only were the 2×2 tables separated for CRP and CRC outcomes, but they were also developed based on the following 2 scenarios.

#### Best Accuracy Model:

When more than one CNN model (and thus more than one 2×2 table) was developed in the primary studies, the model with the highest accuracy measure (per outcome) was selected to be used in this scenario. If only one model (and thus one 2×2 table) was formed in the primary studies, data from that single model were combined with the data from other studies.

#### Worst Accuracy Model:

In contrast to the previous scenario, in this scenario, the model with the lowest accuracy measure (per outcome) was entered into the analysis when more than one CNN model (leading to more than one 2×2 table) was present in the primary research. If only one model (and thus one 2×2 table) was formed in the primary studies, data from that single model were combined with the data from other studies.

The predictive performance measures of the CNN models in the detection of CRP and CRC outcomes were calculated in the 2 scenarios (resulting in four outputs). The accuracy measures estimated and combined based on the 2×2 table data were sensitivity, specificity, PLR, and NLR. Due to the dependence of the PPV and NPV on the prevalence of outcome (frequency of CRP or CRC in the primary studies), they were not entered into the combination data used in the meta-analysis.^47,48^ The abovementioned measures were combined using the “midas” module in STATA.^49^ The summary ROC was also used to combine sensitivity and specificity of primary studies separately for CRP and CRC.^50^

The concordance or C-statistics index was also calculated as a measure of discrimination.^42^ This index was obtained by dividing all items in the 2×2 table that have been correctly identified by the CNN model (all true positive and true negative cases) by the total number of subjects (accuracy measure). Given the proportional nature of this index, the “metaprop” module was used to combine this index in the primary research.^51^ The binomial exact method was also applied to calculate the standard error of proportion.^52^ The “logit” transformation was used to assess the potential factors affecting this index^53^ and the logit-transformed accuracy measure was then entered into the meta-regression model.

Heterogeneity was assessed using Cochran’s Q test and I^2^ index and categorized based on Higgins et al’s recommendations.^54^ Subgroup analysis and meta-regression were applied to assess possible reasons for heterogeneity and changes in the C-statistics index. The publication bias was assessed as described by Deek’s et al.^55^ In case of a significant positive result, the trim and fill method was adopted to more accurately assess publication bias.^45^

Sensitivity analysis using the results of ROB assessment on C-statistics index was performed to determine the robustness of the meta-analysis results. The leave-one-out method was then applied to measure the effects of each primary investigation on the overall combination of studies.^56^

The certainty of evidence was determined using the Grading of Recommendations, Assessment, Development and Evaluations (GRADE). Although the GRADE was originally designed for systematic reviews and meta-analyses of interventional studies, a guide for diagnostic studies and tests was recently published by the original designers of the method (McMaster GRADE Center, McMaster University, Canada). These guidelines were adopted in the present study.^57–59^

## Results

**Figure 1.**
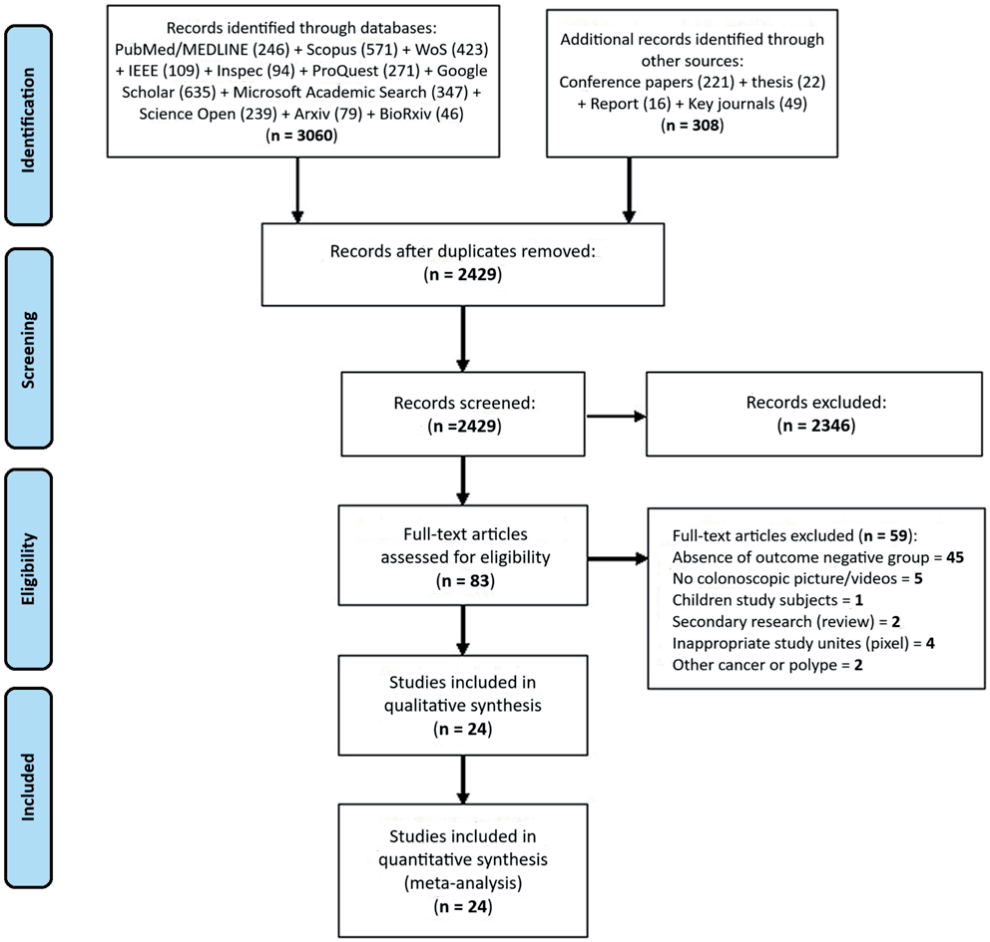
PRISMA flowchart showing the stages from search to selection processes and including primary studies in this systematic review.

A total of 3368 records were extracted from the search conducted in all sources. Of these, 3060 were obtained from official sources (scholarly databases and key journals) and 308 from unofficial sources (i.e., conferences or conference proceedings, theses, and research reports). AK and ARS then assessed the titles and abstracts of the references of the primary studies. During this screening phase, duplicates were eliminated, and 2429 research were evaluated. Ultimately, AK and ARS independently assessed the full texts of 83 primary studies in terms of inclusion and exclusion criteria. At this point, 59 studies were excluded due to the reasons described in Figure 1. Finally, 24 primary studies^60–83^ were found eligible. At last one 2×2 table, obtained from the comparison of the prediction model and the reference test results, was developed for each study. These studies were also included in the quantitative or statistical analysis (meta-analysis).

The features and details of the included primary studies are presented in Supplementary Table 1. These studies collected data from colonoscopy clinics mainly in European or North American countries including the United States, France, Spain, and the Netherlands (n = 14). The location was not reported in one study. The remaining 9 studies were performed in South Korea (n = 5), Japan (n = 2), China (n = 1), and Iran (n = 1). Unfortunately, most studies (except for 2) did not report the number of study subjects and mainly reported the number and resolution of the used images. These images were mostly extracted from the video recordings of colonoscopies performed on patients. Some studies also reported the number and duration of the videos used.

While 16 studies^60,62,63,66–68,70,72,74-77,79-81,83^ used a CNN model for CRP detection, only 3^73,78,82^ adopted the method for CRC detection. Five primary studies^61,64,65,69,71^ used CNN models to detect both CRP and CRC outcomes. Given the different nature of these 2 outcomes and the potential difference in the performance measures of CNN models used for their determination, the results of the primary studies were separately assessed for CRP and CRC outcomes. Thus, from the 21 studies, at least one version of a CNN model was extracted for CRP detection. Moreover, from the 8 studies, at least 1 version of a CNN model was used for CRC detection. It is also noteworthy that the included studies differed in the number of CNN models used for CRP or CRC outcomes. These numbers ranged between 1 and 8 (Supplementary Table 1).

Supplementary Table 2 presents all PROBAST items for assessing ROB in each primary research. Since details of the age and gender of patients undergoing colonoscopy were not reported, the “Participants” component in both ROB and applicability parts was labeled as unclear ROB in almost all primary studies. “Predicting factors” had the best status of methodological quality in both parts. The analysis item also had an unfavorable or high ROB status in 15 studies (about 72%). Generally, only 1 study had a low ROB status for both ROB and applicability parts,^82^ and the rest had either high or unclear ROB status.

Based on the logic of combining the data of 2×2 tables in different CNN models to predict the CRP and CRC outcomes under the best versus worst accuracy scenarios (as described earlier), a total of 4 scenarios can be discussed.

### Convolutional Neural Network Model Prediction for Colorectal Polyps Detection

**Figure 2.**
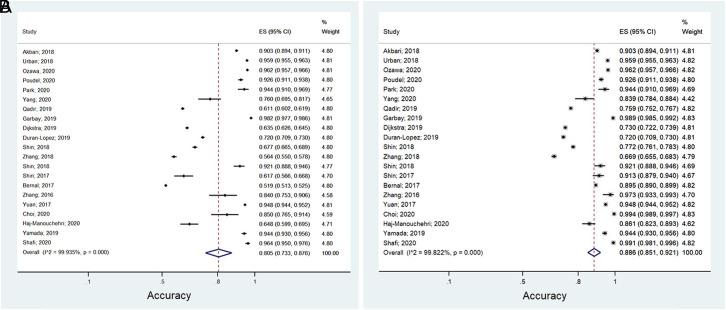
The accuracy measures of CNN models in CRP prediction in the pessimistic (A) and optimistic (B) scenarios. CNN, convolutional neural networks; CRP, colorectal polyps.

**Supplementary Figure 1.**
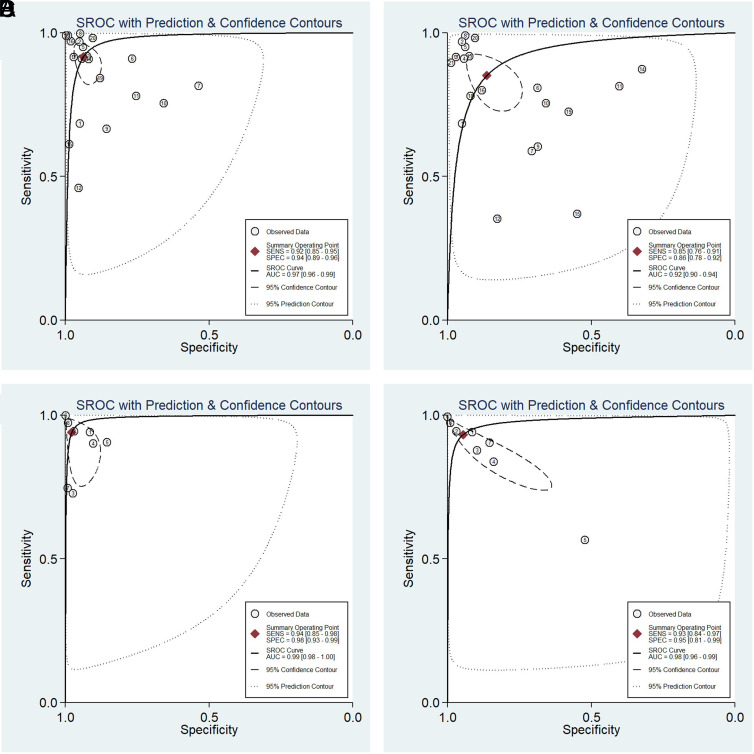
The area under the ROC curve in CRP prediction in the optimistic (a) and pessimistic (b) scenarios. CRP, colorectal polyps.

To predict this outcome, 21 primary studies were used (those reporting at least 1 CNN model for CRP detection). Of these, 8 (38%) only had 1 CNN model and the remaining 13 (62%) had more than 1. The sensitivity and specificity of CNN models in predicting CRP in pessimistic and optimistic scenarios ranged from 84.7% to 91.6% and from 86.0% to 93.8%, respectively (Table 1). The PLR varied from 6.2 to 14.5 in these scenarios. The NLR varied from 0.09 to 0.17 in these scenarios (Table 1). The DOR of CNN models in predicting CRP in pessimistic and optimistic scenarios ranged from 36 to 162, respectively (Table 1). The accuracy measures of CNN models in CRP prediction ranged between 80.5% and 88.6% in the worst and the best scenarios, respectively (Figure 2). The area under the ROC (or AUC index) varied between 0.92 and 0.97 in the worst and the best scenarios (Supplementary Figure 1). The accuracy measures of CNN models for CRP detection are summarized in Table 1.

### Convolutional Neural Network Model Prediction for Colorectal Cancer Detection

**Figure 3.**
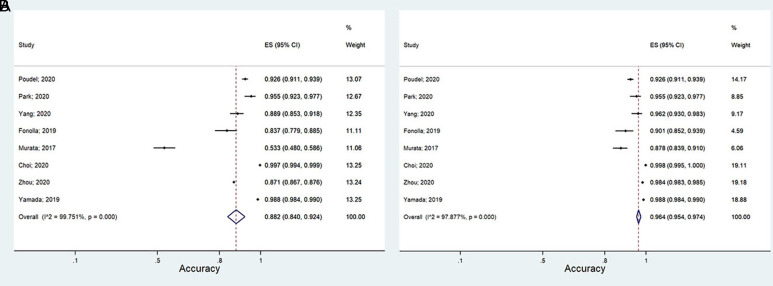
The accuracy measures of CNN models in CRC prediction in the pessimistic (A) and optimistic (B) scenarios. CNN, convolutional neural networks; CRC, colorectal cancer.

Eight primary studies, where at least 1 CNN model was used for CRC outcome prediction, were examined for this purpose. Of these 3 (38%) only had 1 CNN model and the remaining 5 (62%) had more than 1. The sensitivity and specificity of CNN models in predicting CRC in the worst and the best scenarios varied from 93.2% to 94.1% and from 94.6% to 97.7%, respectively (Table 1). The PLR and NLR in the mentioned scenarios were 17.1-41.2 and 0.07-0.06, respectively (Table 1). The DOR of CNN models in predicting CRC in pessimistic and optimistic scenarios ranged from 239.63 to 677.47, respectively (Table 1). The accuracy measure of CNN models for CRC prediction ranged between 88.2% and 96.4% in the worst and the best scenarios (Figure 3). The area under the ROC (or AUC index) varied from 0.98 to 0.99 (Supplementary Figure 1). The accuracy measures of CNN models for CRC prediction are summarized in Table 1.

### Subgroup Analysis in Convolutional Neural Network Model Prediction of Colorectal Polyps and Colorectal Cancer Outcomes

The results of subgroup analysis for CRP and CRC outcomes are summarized in Table 2. While various accuracy measures exist, subgroup analysis in the different scenarios was conducted using the accuracy measure. None of the potential subgroup variables (covariates) affecting the accuracy result (i.e., transfer learning, data augmentation, and methodological quality or ROB) was able to reduce I^2^ index and only data augmentation changed the accuracy measures. The accuracy measures in primary studies with data augmentation were about 10% less than that in studies without data augmentation.

Among factors affecting the accuracy of CRC outcomes, data augmentation was able to change accuracy. However, the difference between studies with and without data augmentation was not as large as that seen in CRP outcome. The methodological quality factor, i.e., ROB, had a significant effect on the accuracy measures of CRC outcome. In other words, groups with low and high ROB had a significant difference in the accuracy measure.

### Meta-Regression Analysis of the Effects of Some Factors on the Accuracy of Colorectal Polyps and Colorectal Cancer Outcomes

**Figure 4.**
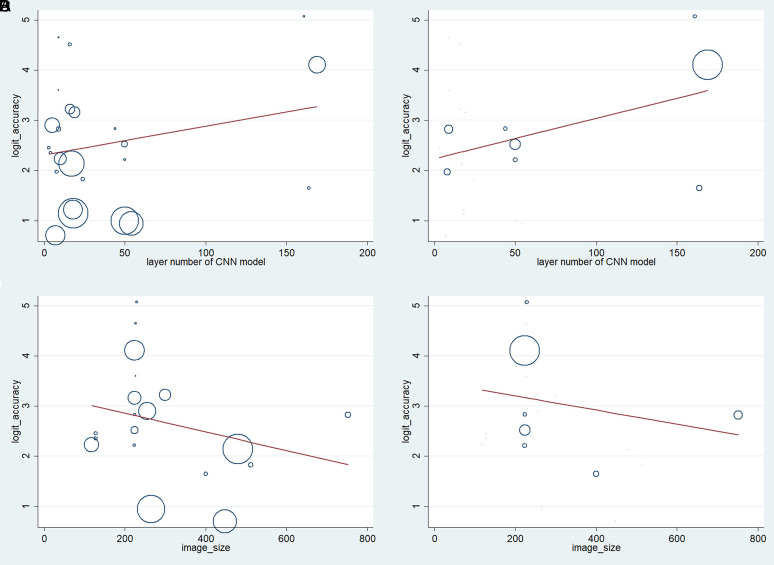
Meta-regression of the effects of number of CNN layers and image size (the number of pixels in the horizontal or vertical axes) on the accuracy of CRP (A and C, respectively) and CRC (B and D, respectively).

The transformed logit of the accuracy measure was used in the meta-regression analysis. The effects of 2 quantitative factors, namely the number of CNN layers and image size (the number of pixels in the horizontal or vertical axes), on the logit of accuracy measures of CRP and CRC outcomes were assessed (Figure 4). The results showed that neither the number of the CNN layers (used in studies) nor image size had a significant relationship with the accuracy measure logit.

### Publication Bias and Sensitivity Analysis

**Supplementary Figure 2.**
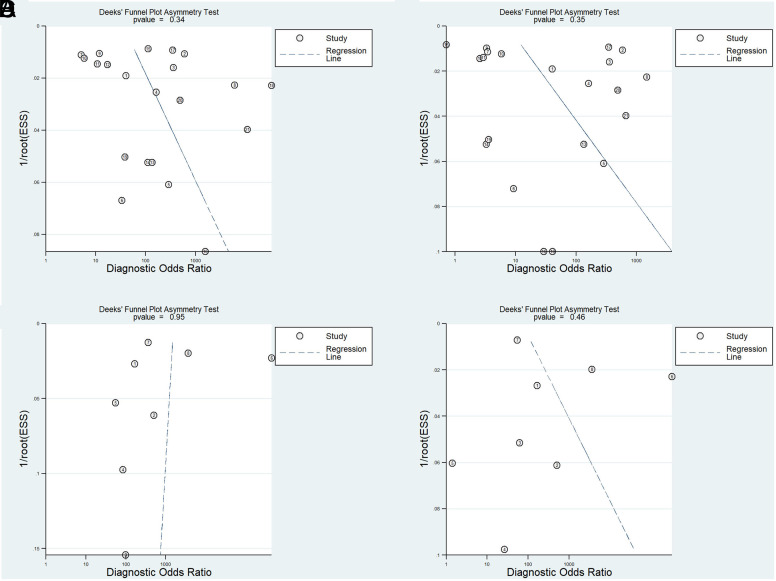
Deek’s funnel plot on the logit of accuracy measure in CRP outcome in the pessimistic (a) and optimistic (b) scenarios and CRC outcome in the pessimistic (c) and optimistic (d) scenarios.

Deek’s funnel plot on the logit of accuracy measure was used to assess publication bias.^55^ No significant publication bias was observed in CRP and CRC outcomes in either the worst or best scenarios (*P* > .10 for all plots; Supplementary Figure 2).

The robustness of the results was assessed using the leave-one-out method in the “Metaninf” module of STATA. No significant difference in the accuracy measures was observed as a result of the presence or absence of any of the studies in the combination of eligible studies.

### Certainty of Evidence

The certainty of evidence (quality of evidence) was assessed based on the GRADE for diagnostic studies. The criteria for downgrading and upgrading factors and their definition are presented in Supplementary Table 3. The results of using this method for CRP and CRC outcomes are summarized in Supplementary Table 4. Generally, the certainty of evidence was at the lowest definable level (very low).

## Discussion

In this systematic review and meta-analysis study, a highly comprehensive search approach in various sources (including databases of various scientific fields and even conference sources) was applied to extract primary research comparing the performance of CNN-based models with the gold standard (i.e., colonoscopy and histological diagnosis by experts). The results showed that CNN models have a high diagnostic value or validity in the detection of colon polyps and malignancies and can be used to identify missed polyps or malignant lesions.

Several recent studies have highlighted the benefits of CNN models for classification purposes. However, due to differences in the classification structure of polyp lesions or malignancies in various primary studies, we failed to collect an acceptable number of homogeneous studies for the meta-analysis of the lesion classification improvement index.

According to the results of the present study, the sensitivity and specificity of CNN models in CRP detection were 85%-92% and 86%-94%, respectively. The accuracy and AUC measures for these models were 81%-89% and 0.92%-0.97%, respectively. These results agree with those reported by systematic review and meta-analysis studies that used the diagnostic value approach.^26,27^ As we could not find any published systematic review and meta-analysis study of diagnostic performance of CNN models in the detection or classification of malignant colorectal lesions, this seems to be the first systematic review to address the matter.

According to the results of the present study, the sensitivity and specificity of CNN models in CRC detection were about 93%-95% and 95%-98%, respectively. The accuracy and AUC measures were about 88%-97% and 0.98%-0.99%, respectively.

Most previous systematic review and meta-analysis studies did not use the diagnostic performance approach for the comparison of CNN models with routine traditional methods of colorectal lesion detection.^22-25,28–32,34,35^ In fact, they generally compared the 2 methods by calculating RR and OR values. Hence, their findings could not be compared to the results of the present secondary studies.

The present systematic review study had strength points including searching at least 10 databases including engineering and computer science databases and conference resources, inclusion of both CRP and colorectal malignant lesions, the use of appropriate ROB assessment tools in the studies of prediction models, and inclusion of primary studies that compared several CNN models derived from CNN models in terms of diagnostic value. It, however, had a number of limitations. First, most included studies used data from archived datasets in some countries. Therefore, although the study subjects (colonoscopy images) were not exactly the same, they were obtained and provided by the same source. This might have increased the overall influence of their results on the findings of this review study. Second, most included primary studies had unfavorable methodological quality. In addition, as patients’ demographic details were not reported in the included studies, comparison of the results and subgroup analysis based on these important variables were impossible. Third, CNN models were technically dependent on the images extracted from colonoscopy videos. While the images used in the included investigations did not belong to just 1 patient, in some studies, they were extracted from as few as 3 video files (probably belonging to 3 patients). This limitation might have falsely increased the accuracy of the results. Fourth, the inconsistency in reporting objectives related to the classification of CRP and CRC lesions seriously limited the possibility of combining included studies. Clearly, given the categorical nature of these lesions (e.g., types of polypus adenomatous lesions, serrated, and so on), they could not be appropriately compared unless the number of categories and their types were consistent in all studies.

Artificial intelligence-based models, particularly CNN-based models, have not been long evaluated in CAD research. Meanwhile, the number of models that use images in the detection of various lesions (such as malignant lesions) is increasing every day. Therefore, further studies are warranted to compare CNN models and other alternative models in terms of diagnostic performance. A recently published systematic review and meta-analysis study^34^ did not limit the methodology of primary studies to AI-based techniques and other techniques such as chromoendoscopy or increased mucosal visualization system (IMVS) were also included. The results showed the relative superiority of AI-based techniques over other techniques, i.e., the relative superiority of CNN models, chromoendoscopy, and IMVS over the standard methods was 7.4%, 4.4%, and 4.1%, respectively.

Based on the results of primary and secondary studies published in the recent 3-5 years, it seems that the methodological quality of primary studies, especially in the field of data sciences and computer engineering (and of course interdisciplinary fields such as medical engineering), has significantly improved. Meanwhile, researchers in this field are recommended to use approved strategies in the design and conduct of diagnostic value and prediction model studies. They are also required to adhere to the writing standards of these studies, including the Standards for Reporting of Diagnostic Accuracy Studies^84^ and the Transparent Reporting of a Multivariable Prediction Model for Individual Prognosis or Diagnosis.^85^ Moreover, in order to increase the validity of the obtained results, it is necessary to perform not only retrospective cross-sectional studies (using archived data available in colonoscopy clinics) but also studies with an ongoing or prospective approach in the settings of colonoscopy centers. While colonoscopists and pathologists use samples collected from colorectal lesions, such studies will be beneficial in determining the efficiency of CNN models in real-time and practical conditions of these clinics.

## Conclusion

Using a relatively comprehensive search of potential sources in various scientific fields, the present systematic review and meta-analysis study showed that CNN-based models could have acceptable accuracy in detecting colon polyps and colorectal malignancies. Their performance would require adequate training dataset using a large number of images extracted from colonoscopy videos. However, since the results indicated the low methodological quality of studies and low certainty or strength of evidence, further primary research with various prospective designs and improved quality are warranted. It is also necessary for researchers to design primary investigations with different subsets of CRP and CRC to determine the diagnostic value or predictive performance of CNN models in classification of these lesions. Such designs will also facilitate the possibility of combining homogeneous results in future systematic reviews and meta-analyses.

## Data Availability:

Data described in the manuscript are available from the corresponding author upon reasonable request.

## Figures and Tables

**Table 1.** Summary of Accuracy Measures of Different Scenarios for Predicting Colorectal Polyps or Colorectal Cancer

Outcome	Scenario	Sensitivity	Specificity	PLR	NLR	AUC	Accuracy	DOR
CRP	Best	91.6	93.8	14.5	0.09	0.97	88.6	162
		(84.7 to 95.6)	(89.3 to 96.4)	(8.4 to 25.2)	(0.05 to 0.16)	(0.96 to 0.99)	(85.1 to 92.1)	(59 to 445)
CRC	Best	94.1	97.7	41.2	0.06	0.99	96.4	677
		(85.2 to 97.8)	(93.5 to 99.2)	(13.7 to 124.2)	(0.02 to 0.16)	(0.98 to 1.00)	(95.4 to 97.4)	(108 to 4240)
CRP	Worst	84.7	86.0	6.2	0.17	0.92	80.5	36
		(75.4 to 90.9)	(77.0 to 91.9)	(3.6 to 10.8)	(0.10 to 0.29)	(0.90 to 0.94)	(73.3 to 87.6)	(13 to 98)
CRC	Worst	93.2	94.6	17.1	0.07	0.98	88.2	240
		(84.0 to 97.3)	(81.1 to 98.6)	(4.3 to 68.4)	(0.03 to 0.19)	(0.96 to 0.99)	(84.0 to 92.4)	(23 to 2483)

AUC, Area under curve; CRC, colorectal cancer; CRP, colorectal polyp; DOR, diagnostic odds ratio; NLR, negative likelihood ratio; PLR, positive likelihood ratio .

**Table 2.** Subgroup Analysis of Some Potential Predictors for Colorectal Polyps or Colorectal Cancer Accuracy

Outcome	Predictors	Levels	No of Studies	Accuracy (95% CI)	*P* ^†^	I^2^ (%)	Test for Interaction
CRP	Data augmentation	Yes	12	84.3 (78.2 to 90.4)	<.001	99.9	0.004
		No	9	94.4 (91.4 to 97.3)	<.001	99.4	
CRP	Transfer learning	Yes	12	87.8 (83.2 to 92.4)	<.001	99.8	0.64
		No	9	89.7 (83.4 to 96.0)	<.001	99.8	
CRP	ROB (outcome item)	Low ROB	16	88.2 (84.2 to 92.2)	<.001	99.8	0.71
		UC/high ROB	5	90.1 (80.6 to 99.6)	<.001	99.8	
CRP	ROB (analysis item)	Low ROB	7	89.5 (83.0 to 96.0)	<.001	99.9	0.74
		UC/high ROB	14	88.2 (83.6 to 92.8)	<.001	99.8	
		High	7	89.5 (83.0 to 96.0)	<.001	99.9	
CRP	Quality	Moderate	9	87.1 (81.5 to 92.7)	<.001	99.7	0.80
		Low	5	90.1 (80.6 to 99.9)	<.001	99.8	
**CRP**	**All studies**	—	**21**	**88.6 (85.1 to 92.1)**	**<.001**	**88.9**	
CRC	Data augmentation	Yes	7	95.7 (94.4 to 96.9)	<.001	98.2	< 0.001
		No	1	98.8 (98.4 to 99.0)	-	-	
CRC	Transfer learning	Yes	7	96.5 (95.5 to 97.6)	<.001	98.1	0.45
		No	1	95.5 (92.3 to 97.0)	-	-	
CRC	ROB (outcome item)	Low ROB	6	97.2 (96.2 to 98.3)	<.001	98.2	< 0.001
		UC/high ROB	2	92.8 (90.8 to 94.8)	-	-	
CRC	ROB (analysis item)	Low ROB	3	98.3 (96.8 to 99.7)	-	-	0.05
		UC/high ROB	5	94.3 (90.6 to 98.0)	<.001	96.7	
		High	3	98.3 (96.8 to 99.7)	-	-	
CRC	Quality	Moderate	3	95.9 (91.5 to 100.0)	-	-	< 0.001
		Low	2	92.8 (90.8 to 94.8)	-	-	
**CRC**	**All studies**	**-**	**8**	**96.4 (95.4 to 97.4)**	**<.001**	**97.9**	-

CRC, colorectal cancer; CRP, colorectal polyp; ROB, risk of bias; UC, unclear.

^†^Calculated from Q Cochrane test.

**Supplementary Table 1.** Characteristics of the 24 Included Primary Studies

Studies	Country	Outcome	Validation	Outcome ascertainment	Development V (P)	Internal V V (P)	Validation model No.
Akbari et al^60^	USA^†^	CRP	I	C + EP	14 (12872)	4 (4702)	1
Urban et al^63^	NR	CRP	I	C + ?	9 (8641)	11 (NR)	5
Ozawa et al^70^	Japan	CRP	I	C + EP	NR (20431)	NR (7077)	1
Poudel et al^64^	South Korea	CRP, CRC	I	C + EP	NR (4715)	NR (2400)	1/1^♣^
Park et al^65^	South Korea	CRP, CRC	I	C + ?	NR (1848)	NR (410)	1/1^♣^
Yang et al^71^	South Korea	CRP, CRC	I + E	C + EP	NR (3442)	NR (386)	2/2^♣^
Qadir et al^67^	Spain, France, USA^†^	CRP	I	C + EP	36 (7466)	23 (18600)	4
Garbay et al^75^	Spain	CRP	I	WCE	NR (5857)	NR (3586)	2
Fonolla et al^78^	Netherland	CRC	I	C + EP	NR (NR)	NR (203)	2
Dijkstra et al^79^	Spain	CRP	I	C + EP	NR (612)	NR (300)	5
Duran-Lopez et al^72^	Spain	CRP	I	C + ?	15 (40604)	3 (7212)	1
Shin et al^76^	Spain, France, USA^†^	CRP	I	C + EP	31 (612)	38 (5631)	4
Zhang et al^66^	USA^†^	CRP	I	C + EP	16 (14532)	18 (17574)	5
Shin et al^78^	Spain, France, USA^†^	CRP	I	C + EP	NR (1525)	NR (366)	1
Shin and Balasingham^74^	Spain, France, USA^†^	CRP	I	C + EP	31 (1525)	44 (366)	4
Bernal et al^83^	Spain, France, USA^†^	CRP	I	C + EP	51 (19608)	52 (17770)	6
Zhang et al^81^	South Korea	CRP	I	C + EP	NR (1780)	NR (150)	3
Yuan et al^62^	USA^†^	CRP	I	C + EP	4 (61007)	2 (11874)	1
Murata et al^73^	Spain	CRC	I	C + ?	NR (1260)	NR (1080)	8
Choi et al^69^	South Korea	CRP, CRC	I	C + EP	NR (2700)	NR (300)	5 / 3^♣^
Haj-Manouchehri and Mohammadi^68^	Iran	CRP	I	C + ?	2 (257)	1 (395)	2
Zhou et al^82^	China	CRC	I	C + EP	NR (137118)	NR (4840)	6
Yamada et al^61^	Japan	CRP, CRC	I	C + EP	NR (464105)	NR (84615)	1 / 1^♣^
Shafi and Rahman^77^	Spain	CRP	I	C + ?	NR (3372)	NR (844)	6

NR, not reported; CRP, Colorectal Polype; CRC, Colorectal Cancer; I, Internal Validation; E, External Validation; C, Colonoscopy; EP, Expert Pathologist; C + ?, Colonoscopy and no information about Pathology; WCE, Wireless Capsule Endoscopy; V, Video number; P, Picture number; ^†^Arizona State; ♣CRP model No.; /CRC model No.

**Supplementary Table 2.** ROB and Clinical Applicability of Included Primary Studies Based on PROBAST Checklist

Studies	Participants	ROB		Analysis	Applicability			Overall	
		Predictors	Outcome		Participants	Predictors	Outcome	ROB	Applicability
Akbari et al^60^	?	+	+	+	?	+	+	?	?
Urban et al^63^	?	+	+	+	?	+	-	?	-
Ozawa et al^70^	?	+	+	-	?	+	+	-	?
Poudel et al^64^	?	+	+	-	?	+	+	-	?
Park et al^65^	?	+	?	-	?	+	?	-	?
Yang et al^71^	?	+	+	-	?	+	+	-	?
Qadir et al^67^	?	+	+	+	?	+	+	?	?
Garbay et al^75^	?	+	-	-	?	+	-	-	-
Fonolla et al^78^	?	+	+	+	?	+	+	?	?
Dijkstra et al^79^	?	+	+	+	?	+	+	?	?
Duran-Lopez et al^72^	?	+	?	-	?	+	?	-	?
Shin et al^76^	?	+	+	-	?	+	+	-	?
Zhang et al^66^	?	+	+	-	?	+	+	-	?
Shin et al^78^	?	+	+	-	?	+	+	-	?
Shin and Balasingham^74^	?	+	+	-	?	+	+	-	?
Bernal et al^83^	?	+	+	-	?	+	+	-	?
Zhang et al^81^	?	+	+	+	?	+	+	?	?
Yuan et al^62^	?	+	+	+	?	+	+	?	?
Murata et al^73^	?	+	?	-	?	+	?	-	?
Choi et al^69^	?	+	+	+	?	+	+	?	?
Haj-Manouchehri and Mohammadi^68^	?	+	?	-	?	+	?	-	?
Zhou et al^82^	+	+	+	+	+	+	+	+	+
Yamada et al^61^	?	+	+	-	?	+	+	-	?
Shafi and Rahman^77^	?	+	-	-	?	+	-	-	-

ROB, Risk of Bias; +, low ROB/low concern regarding applicability; -, high ROB/high concern regarding applicability; ?, unclear ROB/unclear concern regarding applicability.

**Supplementary Table 3.** Components of Certainty of Evidences (CoE) in According to the Downgrading and Upgrading Factors and the Related Criteria Based on GRADE Methodology

Study design	Quality of evidence (score)	Downgrading factors and criteria	Upgrading factors and criteria
Cross-sectional or cohort	High (4)	**Risk of bias:** problem in any 4 factors of PROBAST tool (ROB section): participants, predictors, outcome and analysis Serious: -1	**Accuracy/Discrimintion measure:** AUC measure High accuracy (AUC ≥ 0.90): +1
	Moderate (3)	**Indirectness:** any problem for generalisability of Population & Outcome in the included studies Serious: -1	**Dose-response Relation:** relation between some features of CNN model and accuracy measure such as number of layers, … Positive association in Meta-aregression: +1
Case-control study	Low (2)	**Inconsistency:** the heterogenity measure (I^2^) Serious (I^2^ ≥ 50%): -1	**Assessing plausible prognostic factors:** effect(s) of important determinants of the accuracy measures (sensitivity, specificity, …) using subgroup analysis or multivariable methods Sub-group analysis or MV methods: +1
	Very low (1)	**Impercision:** the uncertain or wide CI of the sensitivity and specificity measures Seriuos: -1	
		**Publication bias:** the findings of Deek’s funnel plot and test Serious (*P* <0.1): -1	

CI, Confidence Interval; AUC, Area Under Curve; MV, Multivariable.

**Supplementary Table 4.** Findings of GRADE Method in According to the Outcomes

Outcome	No of studies	Study design	Downgrading factors					Upgrading factors			Overall
			RoB	Indirectness	inconsistency	Imprecision	Pub. bias	AUC	doze	Covariate	CoE
Prediction of CRP	21	Cross-sectional	Serious	Serious	Serious	Not serious	Not serious	0.92 (0.90 to 0.94)	No association	Not find	Low ⊕OOO
Prediction of CRC	8	Cross-sectional	Serious	Serious	Serious	Not serious	Not serious	0.98 (0.96 to 0.99)	No association	Not find	Low ⊕OOO

ROB, Risk of bias; AUC, Area Under-curve; Pub. bias, Publication bias; AUC, Area Under-curve; CoE, Certainty of Evidence.

**Supplementary Box 1.** PubMed search syntax.

**PubMed Syntax**	(Neoplas*[tiab] OR Tumor*[tiab] OR Cancer*[tiab] OR Malignan*[tiab] OR Carcinom*[tiab] OR polyp*[tiab]) AND (colorect*[tiab] OR Colon[tiab] OR sigmoid*[tiab] OR rect*[tiab]) AND (“convolutional neural network*”[tiab] OR “neural network*”[tiab]) AND 2010/01/01:2020/02/30[dp]
